# Recent Advances in Multifunctional Mechanical–Chemical Superhydrophobic Materials

**DOI:** 10.3389/fbioe.2022.947327

**Published:** 2022-07-13

**Authors:** Qinghua Luo, Jiao Peng, Xiaoyu Chen, Hui Zhang, Xia Deng, Shiwei Jin, Hai Zhu

**Affiliations:** ^1^ Key Laboratory of Catalysis and Energy Materials Chemistry of Education, Hubei Key Laboratory of Catalysis and Materials Science, South-Central University for Nationalities, Wuhan, China; ^2^ China State Key Laboratory of Biogeology and Environmental Geology, Engineering Research Center of Nano-Geomaterials of Ministry of Education, Faculty of Materials Science and Chemistry, China University of Geosciences, Wuhan, China

**Keywords:** superhydrophobic, mechanical stability, chemical stability, multifunctional, durability test

## Abstract

In recent years, biology-inspired superhydrophobic technology has attracted extensive attention and has been widely used in self-cleaning, anti-icing, oil–water separation, and other fields. However, the poor durability restricts its application in practice; thus, it is urgent to systematically summarize it so that scientists can guide the future development of this field. Here, in this review, we first elucidated five kinds of typical superhydrophobic models, namely, Young’s equation, Wenzel, Cassie–Baxter, Wenzel–Cassie, “Lotus,” and “Gecko” models. Then, we summarized the improvement in mechanical stability and chemical stability of superhydrophobic surface. Later, the durability test methods such as mechanical test methods and chemical test methods are discussed. Afterwards, we displayed the applications of multifunctional mechanical–chemical superhydrophobic materials, namely, anti-fogging, self-cleaning, oil–water separation, antibacterial, membrane distillation, battery, and anti-icing. Finally, the outlook and challenge of mechanical–chemical superhydrophobic materials are highlighted.

## Introduction

Nature has incubated many sophisticated superhydrophobic creatures during long-term evolution and natural selection (Sanchez et al., 2005; Liu et al., 2010). Water droplets are spherical on the lotus leaf surface and can roll away the pollution form the surface, which is caused by the chemical composition and special structure of the surface of the lotus leaf. The waterproof composition and microscopic rough structure on the surface of the lotus leaf cause the superhydrophobic phenomenon. This is known as the “Lotus Effect” confirmed by W. Barthlott and C. Neihuis. In addition, many fascinating superhydrophobic phenomena in nature have been uncovered, such as low-adhesion water striders, water-collecting beetles, high-adhesion rose petals, and gecko feet. Inspired by these natural superhydrophobic phenomena, lots of superhydrophobic materials have been developed and used in many fields, self-cleaning (Wang et al., 2022; Jung and Bhushan, 2009; Lou et al., 2020), anti-icing (Lv et al., 2014; Boinovich and Emelyanenko, 2013; Rico et al., 2020; Xie et al., 2022; Zhang et al., 2021a; Yang et al., 2022; Chen et al., 2021; Liu et al., 2019a), anti-fogging (Yoon et al., 2020a; Feng et al., 2021; Sun et al., 2014; Wen et al., 2014), antibacterial (Wu et al., 2016; Wang et al., 2020a; Ma et al., 2020; Ye et al., 2021), fluid drag reduction (Li et al., 2019a; Hu H. et al., 2017; Liu et al., 2019b), liquid separation (Lv et al., 2017; Gu et al., 2019a, b; Chen et al., 2016; Zhang et al., 2022), membrane distillation (Liao et al., 2020; Guo et al., 2021; Ji et al., 2021), fog harvest (Zhu et al., 2016a; Zhu and Guo, 2016a; Zhong et al., 2018), *etc*.

The construction of superhydrophobic materials is based on the combination of micro/nano structures and low surface energy chemicals (Fu et al., 2019; Wang et al., 2020a). The micro/nano structures are vulnerable to mechanical wear and chemical corrosion in practical application (Verho et al., 2011; Milionis et al., 2016; Tian et al., 2016). Once the superhydrophobic surface is worn or impacted by external pressure, the structure collapses and the chemical substances are worn off, causing the hydrophobic properties to be partially or completely lost immediately and cannot be recovered. In addition, the superhydrophobic materials suffer from the degradation induced by UV exposure and chemical reactions with solvents. Therefore, the development of superhydrophobic materials with excellent mechanical durability and chemical stability are highly desired.

In this review, we illustrated the recent development of multifunctional mechanical–chemical superhydrophobic materials. At first, the theories about superhydrophobic surfaces including Young’s equation, Wenzel model, Cassie–Baxter model, Wenzel–Cassie model, “lotus” model, “gecko” model are elucidated. Then, we summarized the improvement in mechanical stability and chemical stability of superhydrophobic surface. Later, the durability test methods such as mechanical test methods (sandpaper abrasion, tape-peeling, knife-scratch, finger wiping, Taber abrasion, impact test) and chemical test methods (solution immersion, UV irradiation, electrochemical) are discussed. Afterwards, the applications of multifunctional mechanical–chemical superhydrophobic materials are elaborated. Finally, conclusion and prospects of multifunctional mechanical–chemical superhydrophobic materials were discussed.

## Theory of Superhydrophobicity

### Wetting Definitions

**FIGURE 1:**
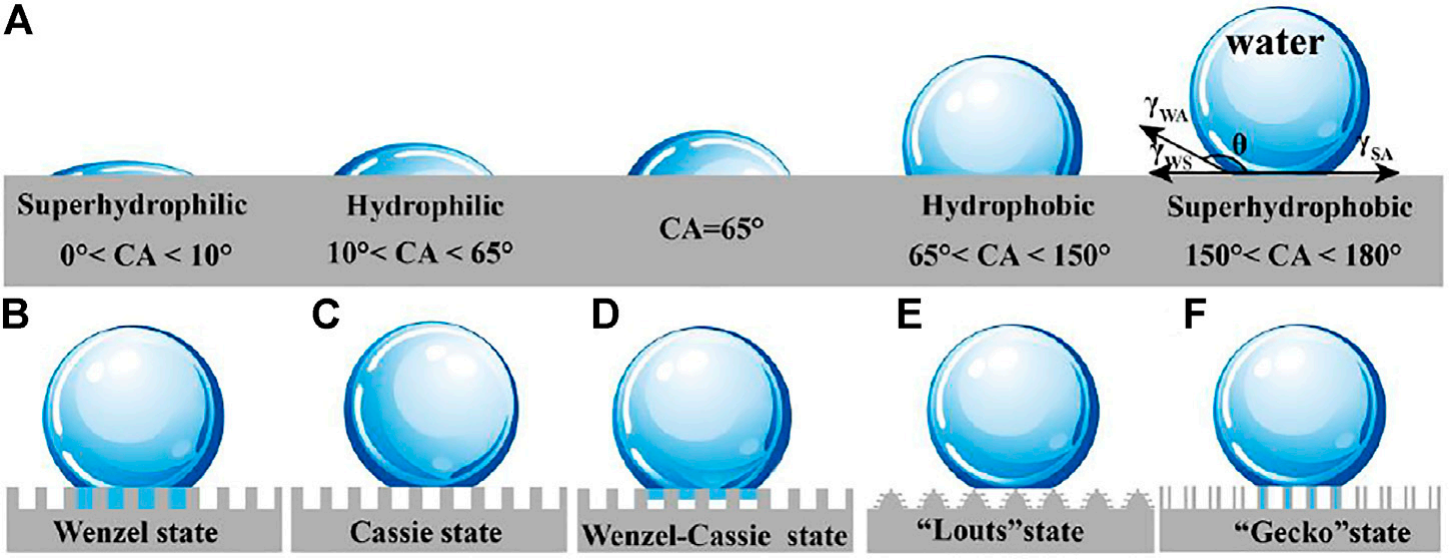
**(A)** Wetting definitions. **(B)** Wenzel model. **(C)** Cassie–Baxter model. **(D)** Wenzel–Cassie model. **(E)** “Lotus” model. **(F)** “Gecko” model (Zhu et al., 2020).

If the interaction between liquid molecules and solid molecules is stronger than that between liquid molecules, the liquid will spread on the solid surface, which is called wetting phenomenon. Wettability is generally characterized by the contact angle of liquid on the solid surface. (Figure 1A) (Tuteja et al., 2007; Xia and Jiang, 2008; Bormashenko, 2019). When water contact angle (WCA) is lower than 10°, the surface is superhydrophilic. And the hydrophilicity is called at 10°–65°, hydrophobicity is denominated at 65° < CA < 150°. Especially, when the WCA is greater than 150°, the sample exhibits superhydrophobicity. Recently, through Jiang’s theoretical research and experimental operation (Xia and Jiang, 2008; Zhu et al., 2021a), it is proved that CA of 65 defines non-wetting and wetting.

### Young’s Equation

In 1805, Thomas Young carried out force analysis on the three-phase interface and proposed a force analysis model called Young’s equation (Young, 1805), which was only applicable to the contact angle value of water droplets with ideal smooth surface when they reached equilibrium state on the surface.

$$\gamma_{\mathrm{SV}}=\gamma_{\mathrm{SL}}+\gamma_{\mathrm{LV}}\mathrm{cos\theta ,}$$

where θ is the static water contact angle; γ_SV_, γ_SL,_ and γ_LV_ represent surface tension of solid–vapor, solid–liquid, and liquid–vapor, respectively.

### Wenzel Model

Based on Young’s equation, Wenzel linked the roughness factor of the surface with the water contact angle by calculating the adhesion force balance in the surface wetting process (Wenzel and Robert, 1936), and the linear relationship between Young’s contact angle and apparent contact angle are acquired:

$$\cos\theta_{w}=\mathrm{rcos\theta}\;,$$

where r is the roughness factor, which is determined by the ratio of the actual surface area to the projected surface area, and θ_w_ and θ represent the water CA in respective apparent and original states.

According to the Wenzel model (Figure 1B), r can be regarded as the amplification factor in a mathematical relationship, which will make the hydrophilic surface more hydrophilic; on the contrary, for a hydrophobic surface, it will make the surface more hydrophobic.

### Cassie–Baxter Model

Cassie–Baxter model (Cassie and Baxter, 1944) can be used to analyze the wettability of porous hydrophobic fabric surface. On the basis of Young’s equation, it is concluded that the apparent contact angle is the sum of the contributions of each contact phase (fabric and air (pore)):

$$\cos\theta_{\mathrm{CB}}=f_{\mathrm{SL}}\mathrm{cos\theta}+f_{\mathrm{LV}}\cos\theta^{\prime }\;,$$

where f_SL_ and f_LV_, respectively, show the fraction between the solid–liquid and liquid–vapor interface at the contacted area and air (f_SL_+f_LV_ = 1). θ_CB_ and θ′ are the apparent contact angle of liquid droplets on rough surface and the contact angle of liquid on ideal air surface (θ' = 180°), respectively. The wetting state described by Cassie is shown in Figure 1C. The droplet is suspended on the convex surface, and the contact area between the surface and the droplet is very small.

### Wenzel–Cassie State

The research of Lafuma and Quéré (Lafuma and Quéré, 2003) shows that Wenzel–Cassie model is an intermediate state between Wenzel model and Cassie model (Figure 1D) where water droplets are semi-filled on solid surface. The Cassie state will transform to the Wenzel state under the stimulation of external energy such as droplet impact, mechanical vibration, and droplet evaporation.

### “Lotus” Model

“Lotus” model (Gao and McCarthy, 2006) is a special Cassie model, lotus leaf surface microscale mastoid and surface wax to give it a repellent ability, These structures (Figure 1E) reduce the contact area between solid surface and liquid, and water droplets are in a semi-suspended state, so pollutants can be rolled away by the falling water droplets, which gives a self-cleaning performance on lotus leaf.

### “Gecko” Model

The “gecko” model (Jin et al., 2005) comes from the classical superhydrophobic nanotube structure, and has good adhesion performance. It is similar to Wenzel model. One is in direct contact with the external atmosphere, and the other is trapped in the nanotube. Due to the change of air volume in the nanotubes, the negative pressure in the nanotubes increases, resulting in high CA, which makes the nanotubes have high adhesion to water (Figure 1F).

## Improvement in the Mechanical Stability

### Self-Hardness

**FIGURE 2:**
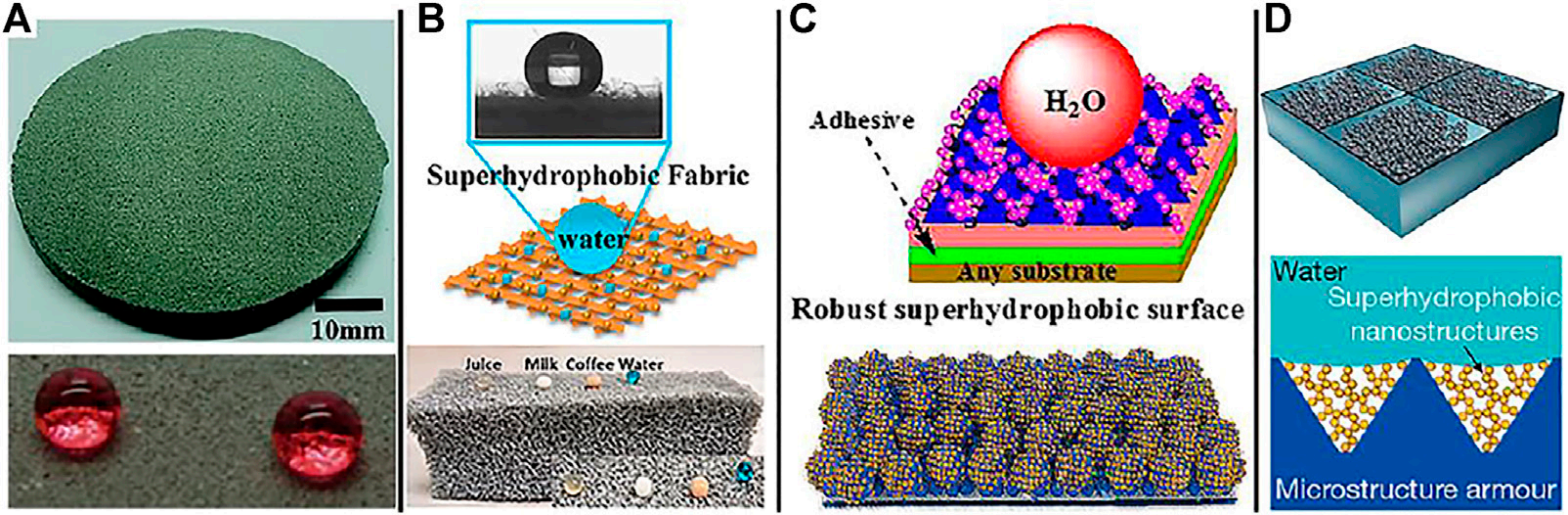
Mechanical superhydrophobic models: **(A)** Self-hardness: the surface of cement (Song et al., 2017a). **(B)** Porous materials: the surface of textile and sponge (Shang et al., 2020; Ozkan et al., 2020). **(C)** “Paint + adhesive” method (Qing et al., 2019; Zhu et al., 2020). **(D)** Schematic diagram of a strategy to enhance the mechanical robustness of superhydrophobic surfaces by containing hydrophobic nanostructures in protective microstructures “armor” (Wang et al., 2020c).

Cement (Song et al., 2017a), diamond (Yang et al., 2014; Wang et al., 2017; Wang et al., 2020b), and alloys (Qiao et al., 2018; Wu et al., 2018) have inherently high hardness and are thus ideal materials to develop superhydrophobic surfaces with an enhanced mechanical robustness. A superhydrophobic concrete (Figure 2A) was prepared by combining metal mesh covering and fluoroalkylsilane modification (Song et al., 2017a). The obtained concrete can retain its superhydrophobic property after a sandpaper wear test (a pressure of 1100 Pa, standard sandpaper of 360#, and abrasion distance of 8 m). In addition, the superhydrophobic concrete is able to endure the knife-scratch and the hammer blow tests. This effectively demonstrates the remarkable mechanical strength of as-prepared superhydrophobic concrete. For its own hard materials, his preparation method is simple and easy to obtain, but because of the lack of materials, it is not suitable for large-scale production.

### Porous Materials

Sponges (Zhu et al., 2013; Cheng et al., 2019a; Dong et al., 2020), textiles (Luo et al., 2021; Zhou et al., 2021), foamed nickel (Hu et al., 2017; Eum et al., 2019; Wang et al., 2021), and other materials (Hou Y. et al., 2015) with multiple layers and porous (Figure 2B), due to their large specific surface area, even if part of the material surface is rubbed off, the material still remains, so it has abrasion resistance and is an excellent superhydrophobic material. Superhydrophobic textiles (Luo et al., 2021) are manufactured by decorating the textiles modified by polydopamine (PDA) with MXene (Ti_3_C_2_T_x_) and then coating with polydimethylsiloxane (PDMS). The obtained superhydrophobic breathable textiles still maintain superhydrophobic properties in the sandpaper wear test (moving 2 cm with traction under the weight of 50 g), which demonstrates the robustness of the superhydrophobic textiles. Porous material is one of the recent research hotspots, which has the advantages of simple operation, low production cost, and suitable for large-scale production, while at the same time, porous materials have been widely used in separation, catalysis, and other fields.

### “Paint + Adhesive” Method

In order to reduce the dependence of superhydrophobic surface on substrate and strengthen the interface bonding force, a strategy of “Paint + adhesive” was developed to prepare superhydrophobic surface. The surface superhydrophobic layer is connected with the substrate by an intermediate layer, which can not only anchor the micro-nano structure on the surface, but also serve as a shielding layer to provide additional protection for the substrate, thus obviously improving the mechanical properties of superhydrophobic surface and preparing durable superhydrophobic surfaces on various substrates. Lu et al. (Lu et al., 2015) proposed a “paint + adhesive” strategy to build a durable superhydrophobic surface for the first time. TiO_2_ nanoparticles modified by fluorosilane, was dispersed in ethanol solution and sprayed on the adhesive-coated substrate. The adhesive can firmly adhere the TiO_2_ nanoparticles (superhydrophobic layer) to the substrates that the obtained superhydrophobic surface shows a water CA of >160° even after wiping with fingers, impacting with water droplets, and 40 cycles of sandpaper abrasion (standard glasspaper, grit no. 240, and moved for 10 cm). Based on the above “paint + adhesive” method, many organic/inorganic adhesives and superhydrophobic materials are used to develop superhydrophobic surfaces with good durability (Figure 2C) (Zheng et al., 2021). The method can improve the binding force between the substrate and the superhydrophobic material, and can be produced on a large scale which has wide selectivity to the substrate. However, the superhydrophobic layer is affected by external mechanical friction or chemical corrosion, and its service life is greatly reduced.

### “Armor”

Armoring strategy is to use materials with excellent mechanical properties to protect the surface micro-nano structures, which is similar to the function of armor. At present, nano-scale armor and microscale armor are mainly used. In 2020, Wang and coworkers (Wang et al., 2020c) fabricated a robust superhydrophobic surface *via* constructing surface texture at two different length scales, including superhydrophobic nanostructures and a microstructure frame (Figure 2D). The microstructure frame is made up of an array of microscale inverted-pyramidal cavities, which can house the superhydrophobic nanostructure and act as a protective “armor” to avoid the destruction of the superhydrophobic nanostructure by abradants. The combination of superhydrophobic nanostructures and the protective microstructure frame ensures that the obtained superhydrophobic surface could tolerate more than 1000 abrasion cycles and even under tape-peeling tests, Taber abrasion tests, and scratch tests. The armor model provides a new idea for the preparation of durable superhydrophobic materials, but it is still in the exploratory stage because of its complex preparation method.

## Improvement in the Chemical Stability

Improving the chemical stability of superhydrophobic surface is also a research hotspot in recent years. At present, the common preparation methods to improve the chemical stability of superhydrophobic surface include chemical etching, spraying, electrochemical deposition, sol-gel method and electrostatic spinning. However, they have their own advantages and disadvantages. (Table 1).

**TABLE 1:** Advantages and disadvantages of different methods.

Method	Advantages	Shortcoming	Large-scale production
Chemical etching	Convenient preparation	High requirements on etching time, soaking time, *etc.*	Yes
	Cheap raw materials		
	High success rate		
Spraying	Easy to control	Poor adhesion short service life	Yes
	Low cost		
	High spraying efficiency		
Electrochemical deposition	Mature technology simple operation	High cost high equipment requirements	No
Sol–gel method	Heat-resistant, low-cost, simple operation	Easy to crack long preparation time	Yes
Electrostatic spinning	How spinning cost many kinds of textiles	Need to be done at high-voltage high energy consumption	No
	Simple operation		

### Chemical Etching

Chemical etching method refers to the preparation of superhydrophobic surface by using the strong corrosiveness of strong acid/alkali solution to construct a micro/nano composite structure on the substrate, which is simple to operate and fast to react. Xu et al. (Xu et al., 2020a) used nitric acid solutions with different concentrations to etch the nickel mold, discussed the importance of etching time and chemical solution concentration, and then copied the surface pattern of the chemical etching template to obtain a large-area micro/nano-structured polydimethylsiloxane (PDMS) film with superhydrophobicity. The film shows superhydrophobicity even under high-strength friction, and also has excellent acid and alkali resistance (excellent liquid repellency even after contacting with 1 M HCl, 1 M NaOH and 1 M NaCl solutions for 96 h), ultraviolet resistance, and optical transparency.

### Spraying

The spraying method uniformly disperses and overlays the raw materials of micro/nanoparticles on the surface of the base material to mode a uniform coating with a certain structure, which is not limited to the shape and size of the base material, simple and convenient to operate, low in cost, and high in coating efficiency. Yokoi et al. (Yokoi et al., 2015) deposited perfluorodecyl trichlorosilane on the surface of alkali-treated polyester, and then sprayed silica modified by fluorosilane on the surface of modified polyester to acquire a transparent superhydrophobic surface. The contact angle of the sample remained above 150° after 100 wear cycles under the pressure of 10 kPa, and the sample had strong repulsion to strong acid and alkali (the contact angle and sliding angle of acidic and alkaline aqueous solutions with pH values ranging from 2 to 14 were measured. The contact angle of all solutions was over 150°, and the sliding angle was less than 15°), which indicates that the prepared superhydrophobic polyester mesh not only had high mechanical strength, but also had good acid and alkali resistance.

### Electrochemical Deposition

Electrochemical deposition (Lee et al., 2021) method refers to the preparation technology of depositing one or more materials on the workpiece surface of the anode, while the cathode undergoes a reduction reaction. She et al. (She et al., 2014) performed electroless nickel plating on the pre-treated AZ91D magnesium alloy and then electrodeposited the nickel-cobalt alloy coating, obtaining a superhydrophobic surface with a contact angle of 167.3 ± 1.3° and a rolling angle of about 1°, and the corrosion current density is three orders of magnitude lower than that of the blank sample, the corrosion rate is about 0.06% of the blank sample, which shows it has better corrosion resistance and pH stability.

### Sol–Gel Method

Sol–gel method refers to the use of highly chemically active compounds as precursors, hydrolysis, and condensation reaction in the liquid phase to form a stable transparent sol system, after polymerization, gel is formed, and then by drying, sintering curing treatment to prepare micro and nano pore structure, so as to give the surface of the material hydrophobic properties. Su et al. (Su et al., 2017) prepared hydrophobic sol by teosilicate ethyl ester and polydimethylsiloxane according to a certain mass ratio. Polyester fabric absorbed sol by immersion and reacted with acid to prepare superhydrophobic polyester surface with good mechanical stability. The prepared superhydrophobic textiles have excellent durability in deionized water, various solvents (the CAs were almost unchanged and still above 150° immersed in deionized water, hexane, hexane and toluene hexane for 168 h), strong acid/alkali solutions (the superhydrophobic textiles still had water repellency after being immersed in HCl solution for 60 h or an aqueous NaOH solution for 48 h) and boiling water/ice water.

### Electrostatic Spinning

Electrospinning (Wan et al., 2022) is a kind of method in which polymer solution forms a jet under the action of high-voltage electrostatic force, and finally one-dimensional nanofibers are prepared. The superhydrophobic surface can be obtained by covering the surface of the substrate with nanofiber membrane and then modifying it with low surface energy substances. It has the advantages of low spinning cost, simple manufacturing device, various kinds of spinnable substances, controllable process, *etc*. Cui et al. (Cui et al., 2018) prepared superhydrophobic anticorrosive coating on aluminum substrate by electrospinning. Polyvinylidenefluoride (PVDF)/stearic acid nanofibers are used to construct micron/nanometer superhydrophobic structures to provide long-term corrosion protection. After corrosion in 3.5% NaCl solution for 30 days, it still had excellent corrosion resistance.

## Durability Test

### Mechanical Durability Test

Inspired by lotus leaves, superhydrophobic surfaces have huge potential applications. However, their practical application is limited by poor durability. When exposed to harsh mechanical or chemical conditions, they can easily lose their functions. Scientists also try to adopt various methods to improve the durability of materials, so we need to establish a test method for superhydrophobic durability. At present, there are many testing methods of superhydrophobic durability, which can be summarized into two aspects: one is mechanical durability test, such as sandpaper abrasion, tape-peeling, knife-scratch, finger wipe, Taber abrasion, and impact test, the other is chemical durability test, such as acid-base test, solution immersion, UV irradiation, and electrochemical corrosion.

#### Sandpaper Abrasion Test

**FIGURE 3:**
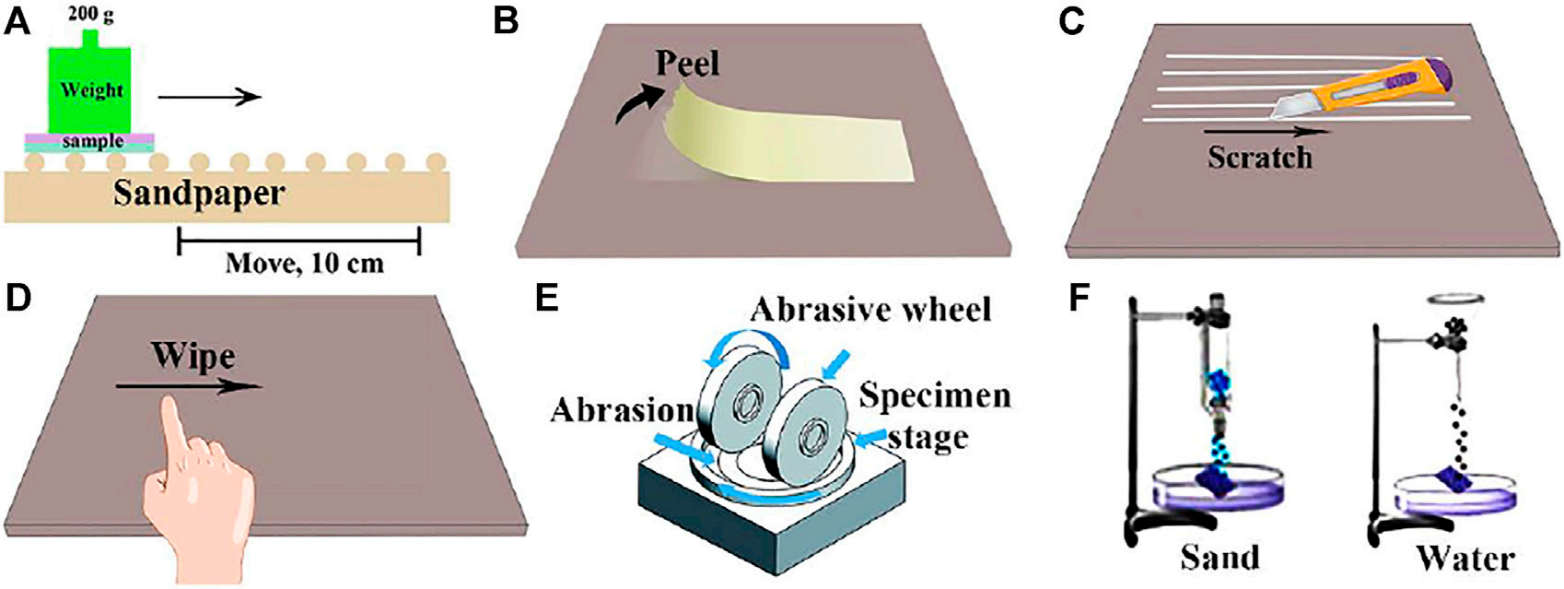
Wear resistance test: **(A)** Sandpaper abrasion. **(B)** Tape-peel test. **(C)** Knife-scratch test. **(D)** Finger wiping test. **(E)** Taber abrasion test (Ye et al., 2017). **(F)** Sand or water impact test.

The sandpaper abrasion test is a common method to test the wear resistance of superhydrophobic surface at present. During the sandpaper abrasion test (Figure 3A) (Zhu et al., 2018a; Wang et al., 2018; Cheng et al., 2019b), a certain load is applied on the superhydrophobic material, and the material is rubbed on the sandpaper. The surface between the superhydrophobic material and the sandpaper acts as a wear surface. Sandpaper abrasion test is the most common evaluation method, which has good practicability. However, at present, the test standards are not uniform and the test error is relatively large. Li et al. (Li et al., 2019b) studied the effects of superhydrophobic coatings prepared with different filler particle sizes on surface morphology and hydrophobic properties under the same load, different abrasive particle sizes and friction distances. The results show that with the same filler content, the larger the filler particle size, the greater the wear resistance.

#### Tape-Peeling Test

Tape peeling (Figure 3B) (Wu et al., 2017a; Zhang et al., 2018; Ghasemlou et al., 2019; Ji et al., 2019) is one of the easiest ways to determine the surface abrasion resistance of superhydrophobic materials, which is to fully contact the tape with the surface of the tested material under a certain pressure, and then peel off at a certain angle and speed. This method is mainly used to test the adhesion strength of superhydrophobic coating and its rough structure to substrate. However, this method can only evaluate the firmness of coating and substrate, but not the strength of superhydrophobic surface, which has certain limitations. By observing the SEM diagram, Zhao et al. (Zhao et al., 2020) compared the number of nanoparticles per unit area before and after peeling, evaluated the binding strength of silica particles with different sizes and epoxy resin substrate, and optimized the superhydrophobic surface durability by adjusting the ratio of different particle sizes to fillers.

#### Knife-Scratch Test

Considering that the superhydrophobic surfaces are often subjected to scratches in practical application, such as car scratches, knife scratch is selected as a typical test to evaluate the mechanical wear resistance of superhydrophobic surfaces. This method is suitable for fields with high requirements for mechanical stability, but the current testing standards are not uniform (Carmalt et al., 2015; Wu Y. et al., 2017; Ghasemlou et al., 2019). As shown in Figure 3C, the knife is used to scrape the superhydrophobic surface, resulting in a dense array of wide and deep scars on the surface. Wu et al. (Wu et al., 2017a) used knives to form wide and deep lattice marks on the superhydrophobic wood, however, water droplets can easily roll down from it without leaving any traces, indicating that the superhydrophobicity still exists.

#### Finger Wiping Test

As shown in Figure 3D, the finger wipe test (Carmalt et al., 2015; Wu et al., 2017a) is to wipe the surface of the superhydrophobic material repeatedly with the finger in the same direction, and then test the change of the contact angle of the material surface. Finger wiping test can preliminarily evaluate the durability of superhydrophobic surface, and the experimental operation is convenient and easy. Liu et al. (Liu et al., 2019a) designed and prepared a new type of polyfluorinated organic superhydrophobic coating based on mercaptan-olefin click reaction. The coating has excellent superhydrophobicity and self-cleaning properties, and has good adhesion to the substrate, which still maintains excellent superhydrophobicity after finger wiping.

#### Taber Abrasion Test

Taber friction (Figure 3E) (Ye et al., 2017; Zhu et al., 2018b) is also a kind of friction test, which is carried out in a special Taber friction testing machine. The machine consists of three parts: a turntable that clamps the sample, a friction wheel and a load. During the experiment, the superhydrophobic material is clamped on the turntable. Then, load a certain weight of the friction wheel for rotating friction, and take out the test piece after the specified number of revolutions to test its superhydrophobic performance. This method has certain evaluation criteria, the experimental operation is convenient and the data is accurate. Peng et al. (Peng et al., 2018) observed the variation of coating contact angle and coating thickness with Taber abrasion cycles under three different loads (150, 200 and 250 g). After 100 wear cycles, the CA of PTFE coating remained above 150° under 150 and 200 g loads and decreased to 146° under 250 g loads.

#### Impact Test

There are two types of impact tests (Figure 3F). One is the water impact test (Zhu et al., 2018b), and the other is the sand impact test (Zhu T. et al., 2020). It is mainly a method to tilt the superhydrophobic surface at a certain angle, impact the surface with sand or water drops at a certain height, and evaluate the change of surface hydrophobicity. This method can effectively evaluate the outdoor durability of superhydrophobic materials. Deng et al. (Deng et al., 2012) used candle soot and silica to prepare superhydrophobic coating. To explore the mechanical properties of the coating, water drop impact and sand wear tests were carried out. Sand particles with a diameter of 100–300 mm hit the surface from a height of 10–40 cm. Although the coating surface is impacted by sand to form a cave, its microstructure has little change.

### Chemical Durability Test

#### Solution Immersion

At present, superhydrophobic materials have been used in various industries; however, their low corrosion resistance hinders their wider application. Therefore, there is a need to, at a relatively low-cost technology, improve the corrosion resistance of these materials. At the same time, scientists used a chemical solution immersion method to test the chemical resistance of materials.

In acidic solution (Si et al., 2015; Zhu et al., 2018a), high concentration of H^+^ will hydrogenate with superhydrophobic materials, which will destroy their original properties and make them lose superhydrophobic properties. In alkali solutions, the chemical properties of strong base are relatively active, with strong reducibility, easy to react with other substances, so as to achieve corrosion. In chloride-containing solutions, because the radius is small and it has strong penetration ability, chloride ions are most likely to pass through the tiny voids in the oxidation film to get to the metal surface, interact with the metal to get soluble compounds, which changes the structure of the oxide film and causes corrosion of the metal. In aqua regia, aqua regia is a very corrosive liquid that can corrode the surface of the material. However, polytetrafluoroethylene (PTFE), the king of organic plastics, is not corroded by aqua regia, so researchers immersed a superhydrophobic material made of polytetrafluoroethylene in aqua regia to test its corrosion resistance.

#### Ultraviolet Light Irradiation

Ultraviolet light irradiation (Zhu et al., 2018a) is one of the common methods for testing the aging of materials, which is mainly tested by putting superhydrophobic materials under a certain wavelength and power ultraviolet lamp, evaluate the attenuation degree of the surface contact angle with the extension of irradiation time. This method is mainly used for evaluating and testing the outdoor durability of superhydrophobic materials. Huang et al. (Huang et al., 2021) used polytetrafluoroethylene (PTFE) particles to prepare powder coatings without solvent and chemical modification. Due to the high bond energy and chemical inertia of PTFE, the surface contact angle of the coating remained above 160° after UV irradiation for 84 h, showing excellent chemical durability.

#### Electrochemical Corrosion

Electrochemical corrosion (Yu et al., 2018) means the corrosion of metal due to electrochemical action in a conductive liquid medium, and current is generated during the corrosion process. When metal is placed in an aqueous solution or in a moist atmosphere, a microcell, also known as a corrosive cell, forms on the surface of the metal, oxidation reaction happens on the anode, so that the anode is dissolved, reduction reaction happens on the cathode, generally only play the role of electron transfer. This method can effectively evaluate the outdoor durability of metallic superhydrophobic materials.

## Applications

### Anti-Fogging

Changing the wettability of the surface is a common method of anti-fogging, and two extreme cases are usually paid attention to: superhydrophilicity and superhydrophobicity. The hydrophilic anti-fogging method, which makes the surface of the substrate highly hydrophilic, the contact angle between the surface of the material and water approaches zero, and makes the water vapor quickly spread on the surface of the substrate after condensation to constitute a transparent water film, which has been deeply studied. Generally, superhydrophobic materials are able to firmly bond with the surfaces of other materials, and water droplets are easy to roll on the superhydrophobic surface. Therefore, it can be inferred that the droplets formed by condensation of water vapor on the surface can also roll off the surface of hydrophobic materials quickly, thus having anti-fogging pe rformance.

**FIGURE 4:**
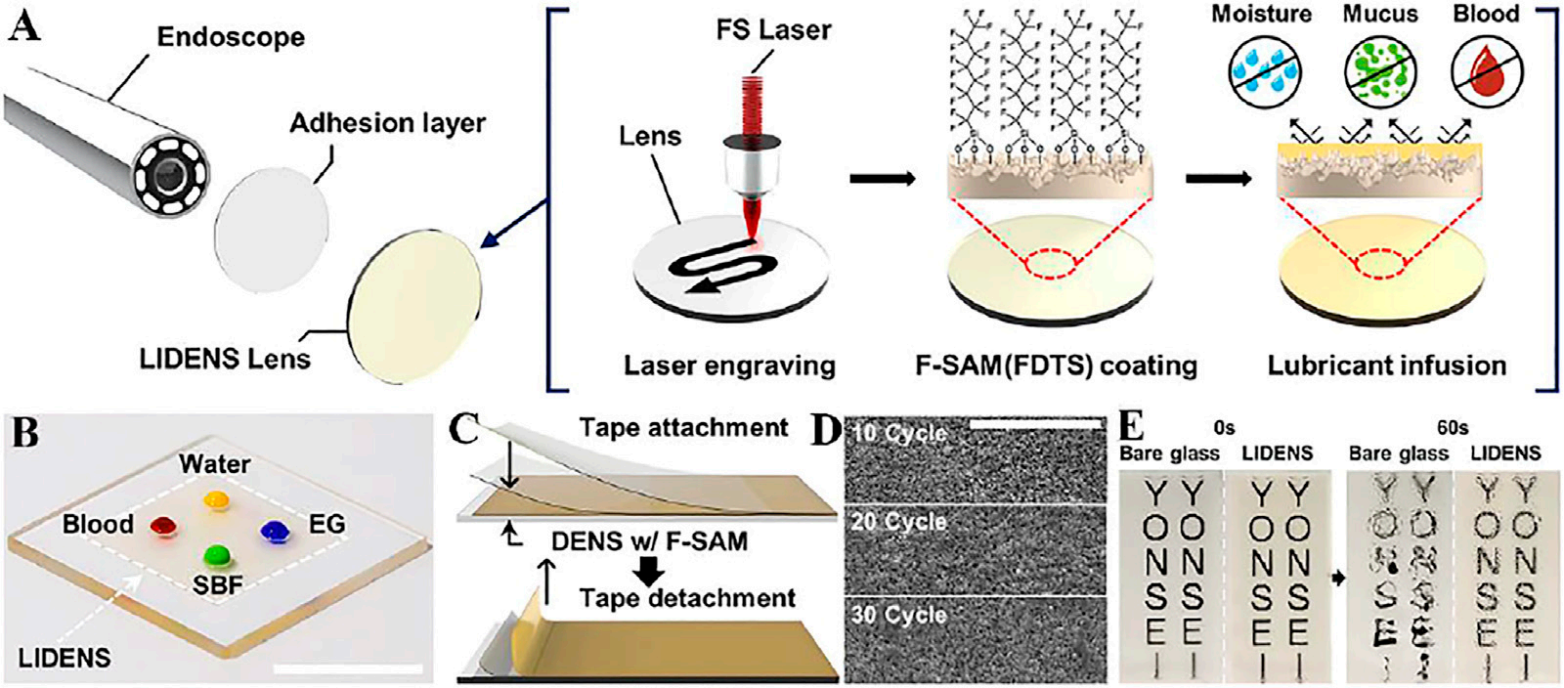
**(A)** Manufacturing process diagram of anti-fogging endoscope. **(B)** Picture of various liquids on the LIDENS (scale bar: 1 cm). **(C)** Schematic diagram of tape-peeling. **(D)** SEM images after 10, 20, 30 tape-peel experiment cycles (scale bars: 20 μm). **(E)** Continuous photographic images after exposed glass (left) and LIDENS (right) are placed on distilled water (∼80°C, 100% relative humidity) for about 3 cm and 60 s (Lee et al., 2020).

Medical endoscopes have promoted the development of medical careers, but endoscopes are prone to mirror fogging due to liquid adsorption and high humidity, which reduces visibility. Lee et al. (Lee et al., 2020) applied a laser to construct a lubricant-infused directly engraved nano/micro structured surface (LIDENS) on the lens, (Figure 4A), which can repel various liquids after chemical modification of the LIDENS lens (Figure 4B). Among them, the injection of lubricant can smoothen the rough surface structure and improve the transmittance. The low cost of LIDENS Nuclear density and dynamic coalescence can remove droplets under gravity, thereby preventing fogging (Figure 4E). At the same time, the mechanical durability of the LIDENS directly etched on the surface morphology was tested, after 30 times of tape peeling (Figure 4C), the SEM images in Figure 3D shows that the dentate wrapped by F-SAM has no obvious topological changes, which proves it has good mechanical properties (Figure 4D).

Yoon et al. (Yoon et al., 2020b) prepared a wet superhydrophobic coating, which maintained excellent anti-fogging performance. The top of the coating is a PDMS micro-well with low surface energy, which shows superhydrophobicity, and the bottom is a sacrificial oil (silicone oil) embedded polymer-silica nanocomposite as hydrophilic part, which guides the upper layer of water vapor condensation to the lower layer. The coating can prevent the formation of fog and maintain optical transparency during condensation.

### Self-Cleaning

The lotus leaves that “come out of silt but do not dye” are typically natural self-cleaning surfaces. In addition, many animals and plants in nature have a superhydrophobic surface with self-cleaning property, such as rice leaves (Bixler and Bhushan, 2012; Nishimoto and Bhushan, 2013; Lee et al., 2017; Xu et al., 2020b), pitcher plants (Song et al., 2017b; Huang et al., 2017; Li et al., 2020a), cicada wings (Oh et al., 2017), butterfly wings (Nishimoto and Bhushan, 2013), gecko feet (Stark et al., 2016), snail shells (Nishimoto and Bhushan, 2013), fish scales (Waghmare et al., 2014), shark skin (Bixler and Bhushan, 2014). Water droplets can capture dust particles and roll away easily when arriving at the superhydrophobic surface, which offers the superhydrophobic surface its self-cleaning property.

**FIGURE 5:**
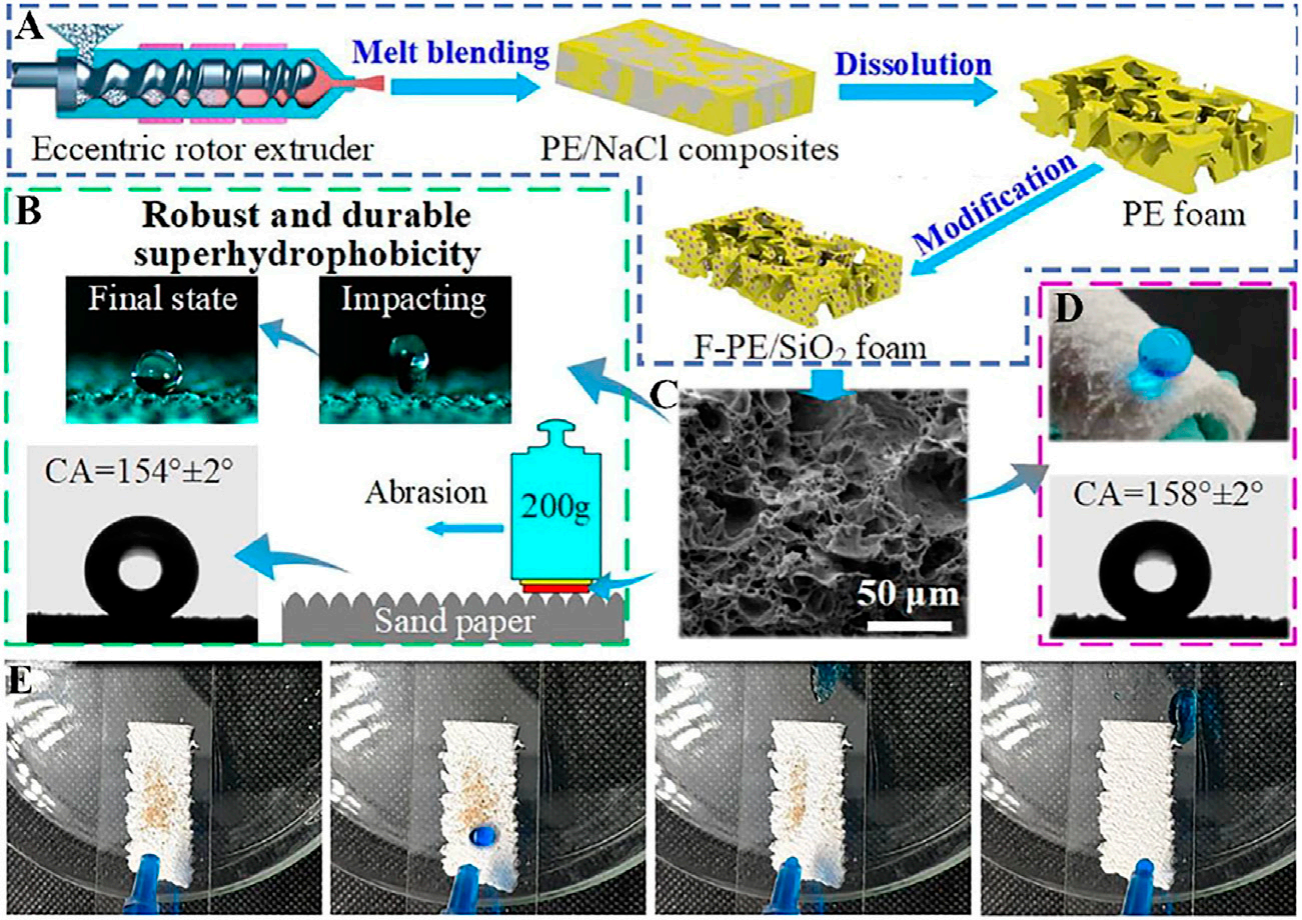
**(A)** PE foam and F-PE/SiO_2_ foam schematic diagram of foam plastic preparation process. **(B)** Illustration of sandpaper abrasion for the foam surface. **(C)** The SEM image of F-PE/SiO_2_ foam. **(D)** Water on the surface of F-PE/SiO_2_ foam. **(E)** Picture of 30° inclined F-PE/SiO_2_ foam polluted by sands before and after water drop washing (Wu et al., 2021).

Wu et al. (Wu et al., 2021) proposed an efficient solution modification method to prepare superhydrophobic F-PE/SiO_2_ foam materials (Figure 5A), which shows a water CA of 158 ± 2° (Figure 5D). The polyethylene foam has an interconnected three-dimensional skeleton, which is composed of a polyethylene skeleton and irregular pores (Figure 5C). The interconnected three-dimensional skeleton results in an enhanced wear resistance for the polyethylene foam. The polyethylene foam still exhibits superhydrophobic property even after sandpaper friction and water impact (Figure 5B). In addition, F-PE/SiO_2_ foam also shows excellent self-cleaning performance (Figure 5E).

Photocatalysis (Liu et al., 2020a; Sutar et al., 2020; Zhu et al., 2021b) can produce self-cleaning effects (Zhu et al., 2020b). Superhydrophobic materials with photocatalytic performance can convert light energy into chemical energy to decompose organic pollutants. During this process, the decomposed organic pollutants leave the surface of superhydrophobic material in the form of gas, and the residual solid particles will be taken away with the spreading of water film.

**FIGURE 6:**
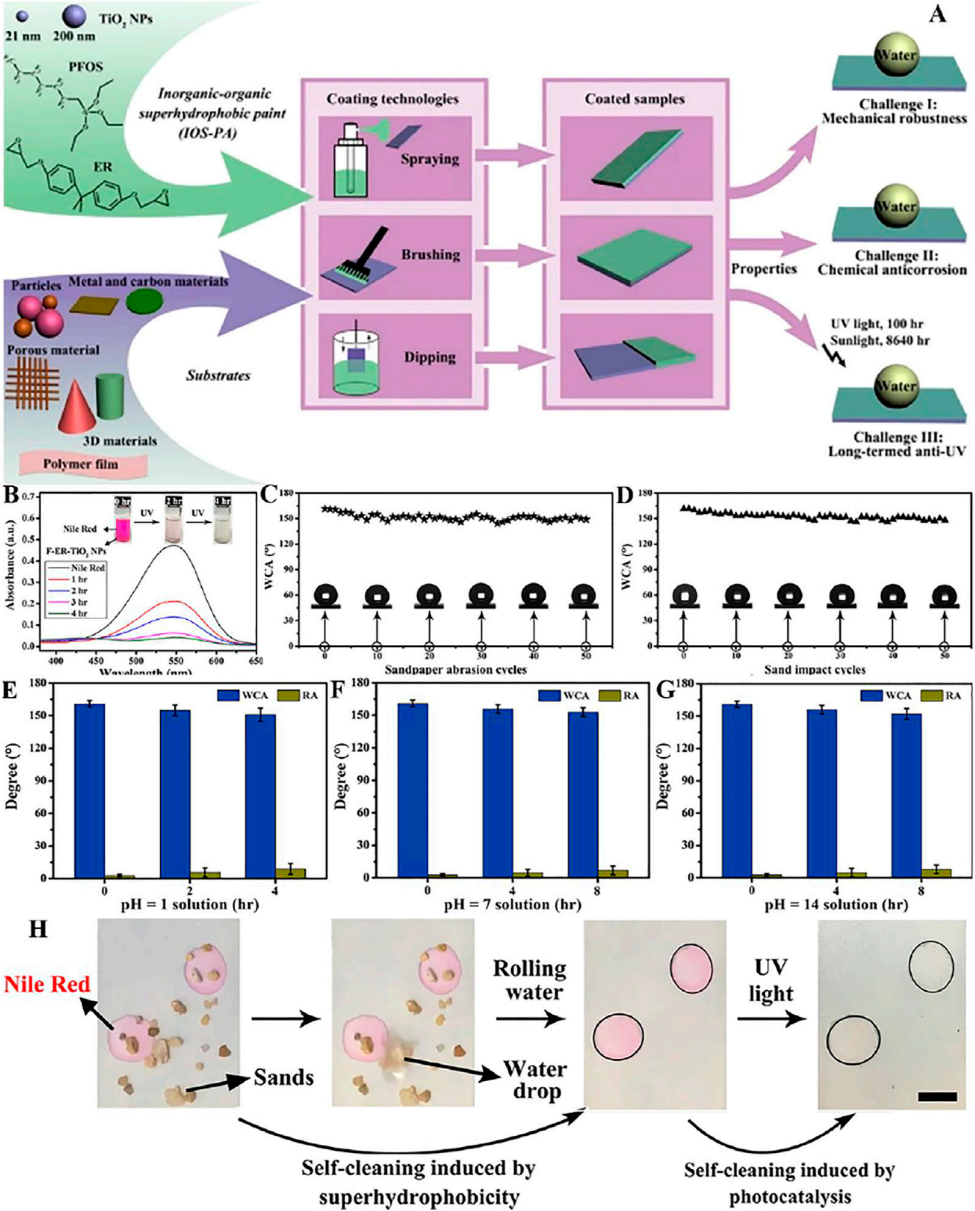
**(A)** Schematic illustration of fabrication of IOS-PA. **(B)** UV–Vis spectra of Nile red solution showing decomposition by F-ER-TiO_2_ NPs every 1  h. The insets are optical photos of the color variations. **(C)** The WCAs of the paint-coated surfaces were tested after each abrasion cycle, and stable superhydrophobicity was obtained with almost all WCAs larger than 150°. **(D)** After sand impact for 50 cycles, the WCAs of the coatings remained high, also showing super water repellency. When placed in pH = 1 **(E)**, pH = 7 **(F)**, and pH = 14 **(G)** solutions for 2, 4, and 8  h, respectively, the coating still manifested super water repellency with high WCAs and low RCAs. **(H)** Multifunctional self-cleaning was shown on the coating, where sand particles could be removed by rolling water, and organic dye could be decomposed by UV light (Zhu et al., 2021c).

Our team (Zhu et al., 2021c) mixed TiO_2_ NPs, epoxy resin and 1H,1H,2H,2H-perfluorooctyltriethoxysilane (FAS) through stirring and ultrasonic treatment to compose an inorganic organic superhydrophobic coating (IOS-PA) (Figure 6A). The presence of TiO_2_ NPs enables the degradation of Nile red (Figure 6B). The superhydrophobicity of IOS-PA is preserved after sandpaper abrasion (Figure 6C) and sand impact (Figure 6D), indicating the excellent mechanical durability. At the same time, after being stored in acidic (pH = 1) solution for 4 h and saline (pH = 7) and alkaline (pH = 14) solutions for 8 h, the high WCA and low RA remained on the coating samples (Figures 6E–G). Moreover, the layer we studied had multifunctional self-cleaning ability, which can not only remove stains by gravity rolling of water, but also decompose organic dyes by ultraviolet (Figure 6H).

### Oil–Water Separation

Frequent oil spills cause serious global water pollution (Liu et al., 2017; Zhu et al., 2020c; Huettel, 2022), which poses an urgent need for efficient solutions to oil–water separation. The traditional methods for oil–water separation include gravity separation (Saththasivam et al., 2016), filtration, centrifugation (Liu et al., 2018), flotation (Rocha e Silva et al., 2018) and electrochemical methods (Kwon et al., 2010). However, most of them have low separation efficiency and complicated operation (Wang et al., 2019). Superhydrophobic material has high separation speed and efficiency and is a promising way to solve this serious matter (Zhu and Guo, 2016b; Kong et al., 2022).

**FIGURE 7:**
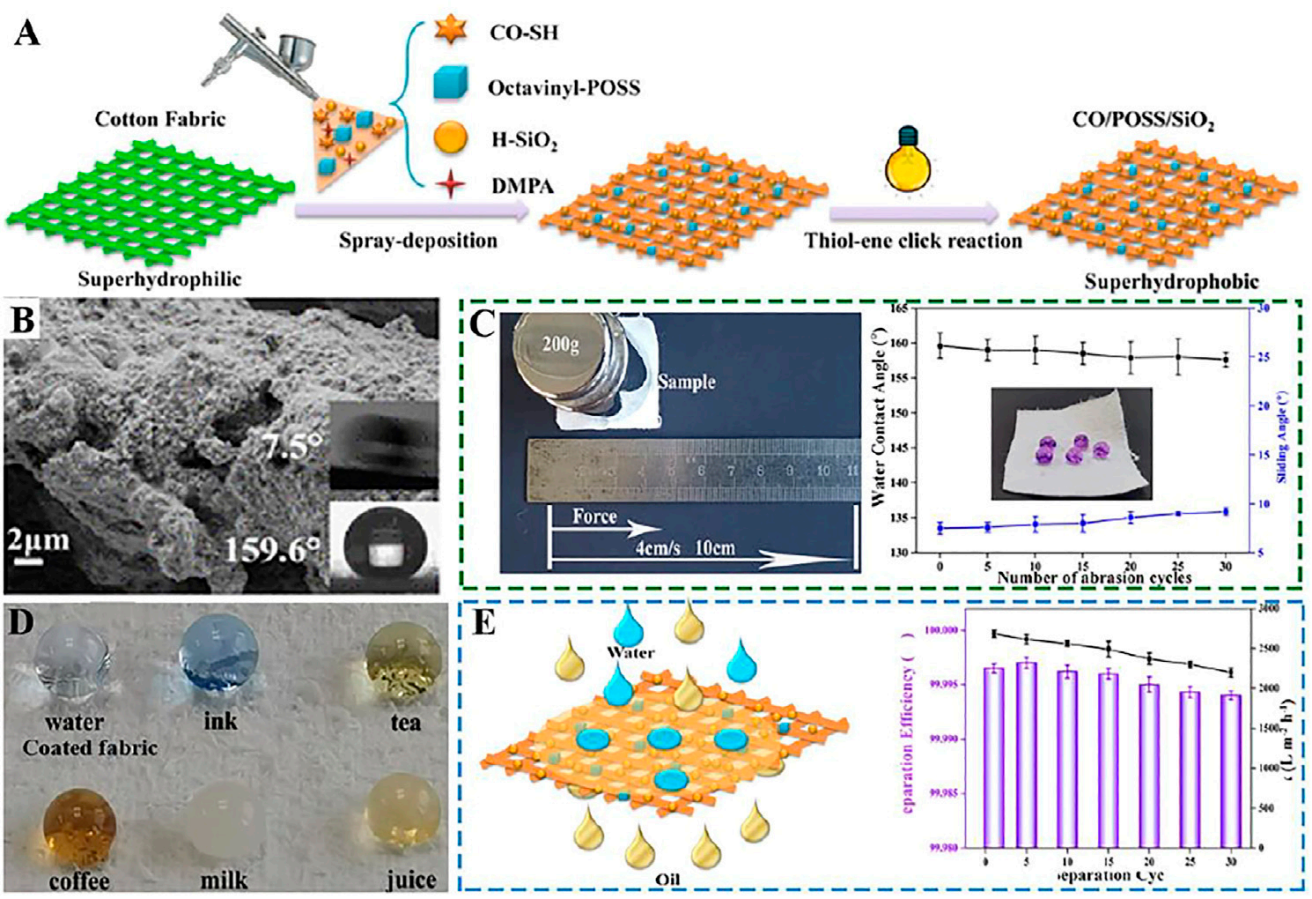
**(A)** Schematic diagram of superhydrophobic cotton fabrics prepared by spray deposition of the thiol−ene resin and UV curing. **(B)** SEM images of the superhydrophobic CO/POSS/SiO_2_ coated cotton fabric and the insets are the corresponding WCA and SA. **(C)** Schematic illustration of the sandpaper abrasion and CA and SA changes after different separation cycles. **(D)** Photos of different liquids on coated fabrics. **(E)** Schematic illustration of the separation process of the oil/water mixture and separation efficiency and flux of petroleum ether/water mixture after different separation cycles (Shang et al., 2020).

Shang and his team (Shang et al., 2020) have prepared an environmentally friendly and sustainable superhydrophobic or superoleophilic castor oil-based nanocomposite on cotton fabric using a thiol-ene chemical method initiated by ultraviolet light (Figure 7A). The cotton fabric has a rough surface and possesses a water CA of ∼160° and a water SA of 7.5° (Figure 7B). The water droplets can penetrate into the pristine fabric immediately because of the capillary effect which is caused by the porosity and abundant hydroxyl groups on the fabric (Figure 7D). In addition, high-strength superhydrophobic cotton fabrics can withstand at least 30 sandpaper wear cycles without losing their superhydrophobicity (Figure 7C). At the same time, the functional cotton fabric can separate kinds oil and water mixtures and emulsions with high separation efficiency (Figure 7E).

Tang et al. (Tang et al., 2021) proposed a cheap, environmentally friendly and pollution-free method to prepare superhydrophobic calcium carbonate (CaCO_3_) which coated stainless steel mesh (SSM). In the experiment, the superhydrophilic CaCO_3_-SSM was firstly prepared by using the biomineralization method induced by bacteria, and immersed in stearic acid (SA) to obtain a superhydrophobic SA/CaCO_3_-SSM. This has regular and large-size micro-pores, and thus shows high oil flux to various oil/water mixtures (0.2–9.12 × 104 L m^−2^·h^−1^) and high efficiency in separation (>94.8%). In addition, the SA/CaCO_3_-SSM also exhibits outstanding wear resistance.

Zhou et al. (Zhou et al., 2016) modified the interior of the PU sponge using (3-mercaptopropyl) trimethoxysilane and graphite oxide by solvent heat treatment, resulting in a graphene layer resembling a crater that was firmly attached to the polyurethane skeleton. Graphene/PU sponges are superhydrophobic with a WCA of over 160° and can effectively separate oil and water.

The recent development of superhydrophobic materials provides a simple and inexpensive solution for oil-water separation. For example, Tudu and Kumar (Tudu and Kumar, 2019) use TiO_2_ nanoparticles and perfluorodecyltriethoxysilane (PFDTS) to make superhydrophobic steel and copper mesh. Yan’s group (Yan et al., 2020) prepared superhydrophobic cotton fabric by combining micro-nano-binary structure of polydopamine (PDA) with grafting of octadecyylamine (ODA).

### Antibacterial Action

The adhesion and proliferation of bacteria on the surface of objects will lead to the formation of biofilms, which poses huge challenges for medical, health, and industrial applications (Monteiro et al., 2022). The antibacterial material based on superhydrophobicity is an emerging method recently (Li S. et al., 2020; Lan et al., 2021). The information of bacterial biofilm involves transportation, adhesion, firmness, and reproduction. The strategies to remove biofilms on the surface of substrates mainly include preventing bacteria from adhesion (Chung et al., 2012) and killing bacteria that have attached.

**FIGURE 8:**
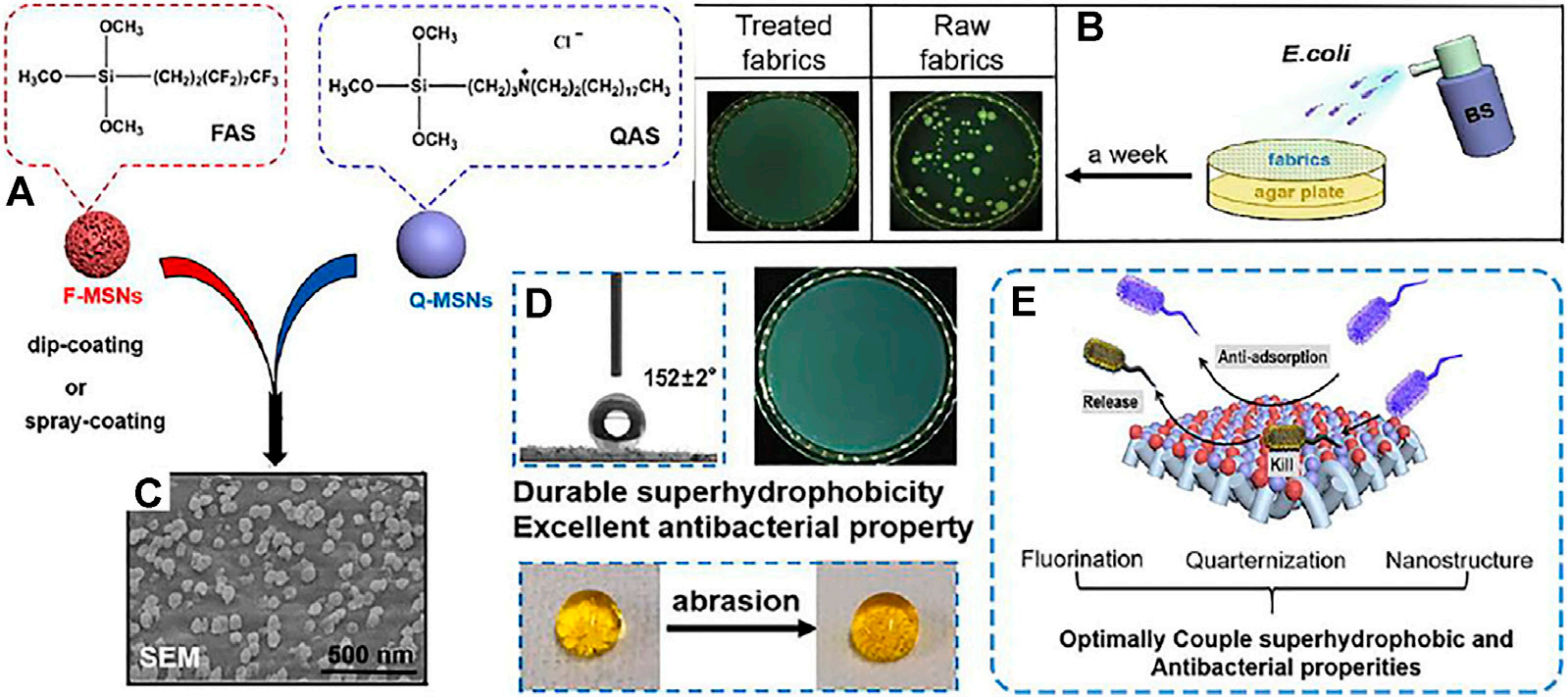
**(A)** Schematic illustration of the configuring process of functionalized textiles. **(B)** Bacterial shielding experiments of cotton fabrics. **(C)** SEM images of the textiles. **(D)** Picture of a water drop (10 μl) on the treated cotton fabrics surface before and after 600 abrasion cycles. **(E)** The schematic diagram of anti-bacterial action (Ye et al., 2021);

Ye et al. (Ye et al., 2021) used PDMS as the adhesive to attach fluorinated mesoporous silica nanoparticles (F-MSNS) and quaternary ammonium functionalized microporous silica nanoparticles (Q-MSNS) (Figure 8A) to the surface of various fabrics (Figure 8C), and the resulting textiles showed obvious synergistic antibacterial effects against Escherichia coli and Staphylococcus aureus by “repellent” (Figure 8B), which is mainly because the superhydrophobicity can repel most bacteria, and Q-MSNS on the surface of cotton fabric can effectively kill some bacteria (Figure 8E). At the same time, due to the surface of F/Q-MSNS coating being rough, even after 600 times of friction, the surface of the coating is still superhydrophobic (Figure 8D).

Ou et al. (Ou et al., 2016) selected polydopamine as an adhesive to prepare a superhydrphobic cotton coated with silver nanoparticles. The polydopamine can increase the binding between silver cotton fibers and nanoparticles, so as to prevent silver nanoparticles from dropping from the surface of cotton fibers. At the same time, the fabric composite has obvious antibacterial effect on Staphylococcus aureus and Escherichia coli.

Zhu et al. (Zhu et al., 2021d) prepared a superhydrophobic coating solution by dispersing hydrophobic silica nanoparticles (Aerosil^®^ gaseous silica) in ethanol at a concentration of 2.5 w/w%. Compared with the bare surface, the attachment amount of SARS-CoV-2 on the superhydrophobic (SHPB) surface was significantly reduced, up to 99.99995%. This suggests that the as-prepared coating can effectively resist the adhesion of severe acute respiratory syndrome coronavirus 2 (SARS-CoV-2) by repelling virus-carrying droplets.

### Membrane Distillation

Membrane distillation (MD) (Laqbaqbi et al., 2017; Hong et al., 2022) is a bright desalination technology because it is capable of treating highly saline water. Deng et al. (Deng et al., 2019) created a unique bilayer composite membrane using the superhydrophobic selective skin of amorphous polypropylene (APP) and the support composition of electrospun polyvinylidene fluoride (PVDF) nanofibers. The permeable vapor flux of the superhydrophobic APP/PVDF membrane is 53.1 kg/(m^2^•h), and the permeable conductivity is stable. At the same time, it has great applicability in MD desalination.

Lu et al. (Lu et al., 2016) developed a porous polyvinylidene fluoride (PVDF) three-porous hollow fiber membrane with superhydrophobicity. The three-pored hollow fiber has greater mechanical strength than traditional single-pored fibers. Under the supreme coating conditions (0.025 wt% Teflon^®^ AF 2400, 30 s), a superhydrophobic surface was obtained which contact angle is 151°. At the same time, Teflon^®^ AF 2400-coated membrane has higher stability, which average flux is 21 kg m^−2^ h^−1^ and rejection rate is 99.99% in 60°C desalination applications.

**FIGURE 9:**
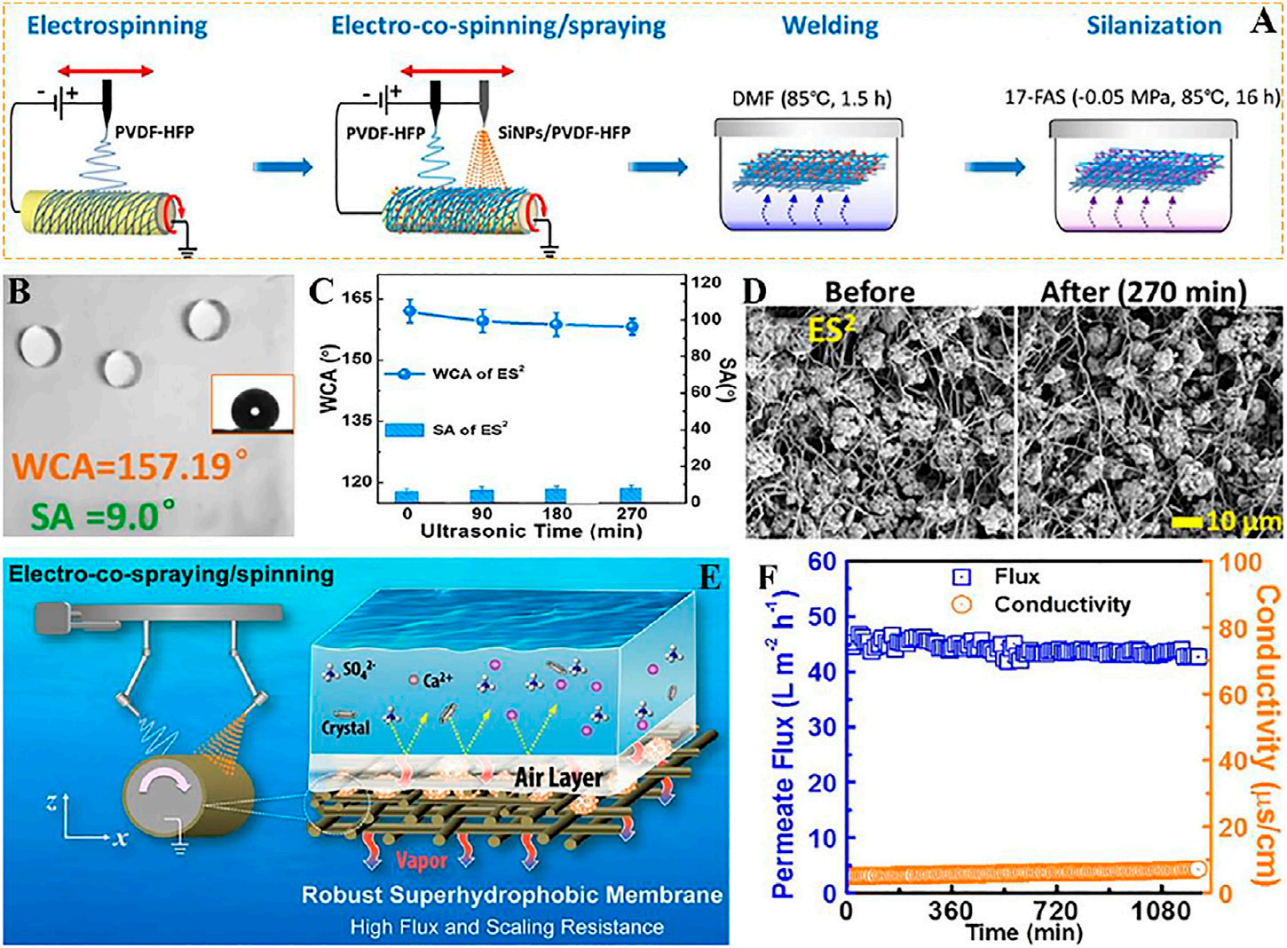
**(A)** Schematic diagram of the ES2 process for fabricating robust superhydrophobic membrane. **(B)** The WCA and SA of robust superhydrophobic membrane. **(C)** WCA and SA of the r-SH membranes after different durations of ultrasonication. **(D)** SEM surface morphology of ES2-derived robust superhydrophobic membrane 270 min before (left) and after (right) ultrasound. **(E)** The mechanism of membrane distillation. **(F)** Vapor flux and conductivity of superhydrophobic electrospun fiber membrane (Su et al., 2019).

Distilled water is produced by the differential partial pressure of steam due to the different temperatures between hot brine and cold deionized water, which drives the transfer of steam from the feed stream to the distillate stream (Figure 9E). Su et al. (Su et al., 2019) used electronic co-spinning/spraying (ES2) with chemical vapor welding to produce superhydrophobic films with mechanical strength, high porosity and robustness (Figure 9A), which also has outstanding vapor permeability (Figure 9F). The prepared superhydrophobic film WCA is bigger than 150° and SA is lower than 10° (Figure 9B). Compared with the superhydrophobic film deposited on the surface of fluorinated nanoparticles, the superhydrophobic film has stronger wettability and wear resistance on MD, the surface of WCA and SA has little change after different ultrasonic treatment time (Figure 9C), and the surface morphology of the solid superhydrophobic film does not change greatly after observation on SEM (Figure 9D).

### Battery

Solar cells (Hegazy, 2001; Liang et al., 2020) are popular because of their low-cost, friendly environment, and renewable characteristics (Syafiq et al., 2018). However, in practical application, the solar cells will affect the efficiency due to the influence of environmental temperature, dust, and wind speed. Therefore, we need to develop a solar cell board which can resist pollution. Superhydrophobic materials can be used in batteries on account of their low surface energy and surface roughness, and they have the characteristics of self-cleaning.

**FIGURE 10:**
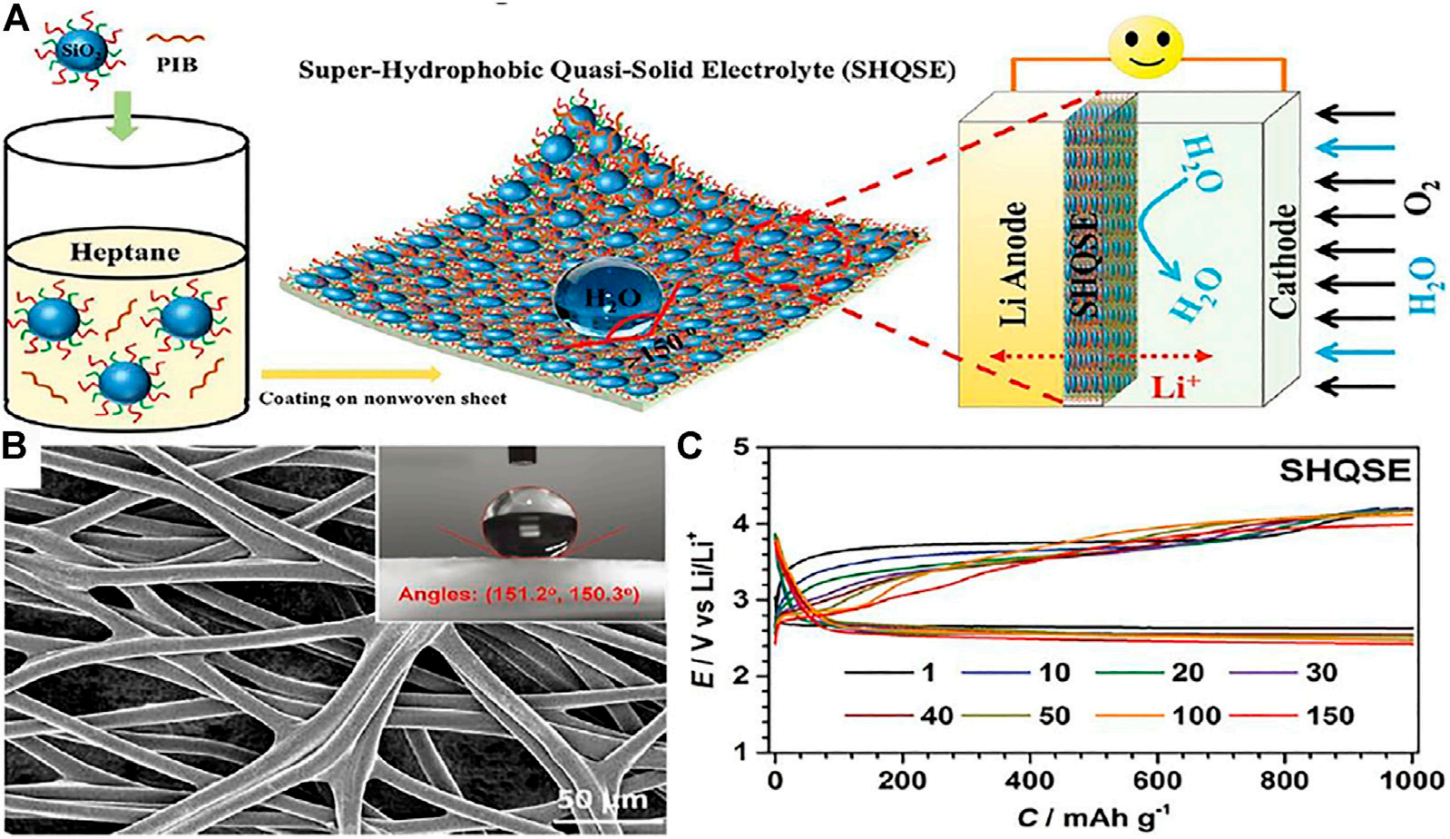
**(A)** Schematic diagram of solid Li-O_2_ battery in humid atmosphere on basis of the superhydrophobic quasi-solid electrolyte (SHQSE). **(B)** SEM image of the original nonwoven fabric and the insets are the corresponding water CA. **(C)** The typical discharge–charge profiles of Li-O_2_ batteries when relative humidity is 45% (Wu et al., 2017b).

Wu et al. (Wu et al., 2017b) developed a viable lithium-O_2_ battery with lithium metal negative electrode in a humid environment (relative humidity of 45%), which prevents H_2_O by constructing a superhydrophobic quasi-solid electrolyte (SHQSE) (Figure 10A). In Figure 10B, the water contact angle is larger than 150°, which indicates that the SHQSE membrane is superhydrophobic and the SHQSE membrane has mechanical stability due to the porous substrate of nonwoven fabrics. From Figure 10C, it displays the classic discharge and charge profiles during cycles, which shows that the hydrophobic effects may take a vital part in the achievement of safe and permanent Li-air battery.

Liang et al. (Liang et al., 2020) used plasma-improved chemical vapor deposition (PECVD) to prepare SiO_2_ as the bottom layer, and then hydrolyzed and condensed epoxy propylpropyltrimethoxysilane (KH560) at both ends to shape a network structure as an intermediate connecting layer. The hydrophilic SiO_2_ modified by hexamethyldisilazane (HMDS) to obtain the top superhydrophobic layer. The structure of the superhydrophobic surface is like the double layer structure of phospholipid in the cell membrane. Compared with the bare glass panel, the glass cover plate used in solar cells greatly improves the efficiency of utilization.

Zhi et al. (Zhi and Zhang, 2018) first formed three-dimensional nanopores crosslinked network by the volatilization of pore-forming agents during calcination, then make the silica nanoparticles attached on the pore structure is formed on the double scale structure, thus forming a kind of superhydrophobic coating, a coating made of surface display WCA is 157.9°, which method is simple, and low coating can be applied in the solar cell cover glass.

### Others

The principle of superhydrophobic anti-icing (Maitra et al., 2014; Boinovich et al., 2015; Liu et al., 2020b) is to cut down the contact area between water drop and the superhydrophobic surface, and postpone the frozen time of water droplets on the surface. Meanwhile, before freezing, water droplets slide down with the help of gravity, reducing the possibility surface icing.

**FIGURE 11:**
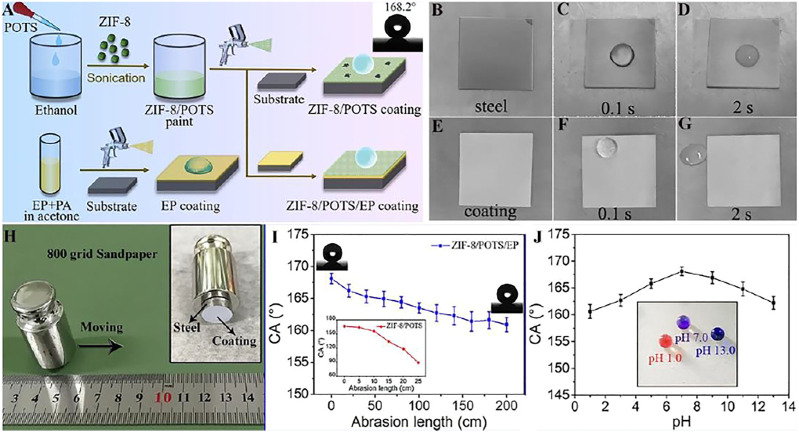
**(A)** The manufacturing process of EP coating, superhydrophobic ZIF-8/POTS coating and ZIF-8/POTS/EP coating. The pictures of **(B**–**D)** Q235 steel sheet and **(E**–**G)** ZIF-8/POTS/EP coating after **(B**,**D)** 2 h in −20°C refrigerator, and after **(C**,**F)** 0.1s and **(D**,**G)** 2 s of dripping 0°C water droplets on their surfaces. **(H)** The schematic of sandpaper abrasion test, and **(I)** the change of abrasion length on the CA. **(J)** The change of pH values of water droplet on the CA of ZIF-8/POTS/EP coating, inset picture is the photograph of litmus colored water droplets with different pH value on ZIF-8/POTS/EP coating (Chen et al., 2021).

Chen et al. (Chen et al., 2021) structed a superhydrophobic composite coating on the basis of MOF (ZIF-8) nanoparticles and organic resins, which shows superhydrophobicity and the water contact angle is 168.2° because of the rough structure of ZIF-8 nanoparticles and the low surface energy (Figure 11A). After being rubbed with sandpaper or immersed in different pH value (Figures 11H–J), the superhydrophobicity can still be maintained, showing that the coating has excellent wear resistance and chemical stability. Figures 11B–G shows the freezing process of the coating surface after dripping 0°C water and the results reveal that the ZIF-8/POTS/EP superhydrophobic coating exhibits great anti-icing properties.

A superhydrophobic surface with a low rolling angle helps to reduce the resistance of the water surface, and the existence of the surface microstructure can make the liquid flow through the superhydrophobic surface to form a gas-liquid two-phase flow, resulting in a slip flow phenomenon, reducing the velocity gradient on the boundary surface, thereby reducing the resistance of the liquid flowing through the solid surface (Venkateshan et al., 2016; Zheng et al., 2020).

**FIGURE 12:**
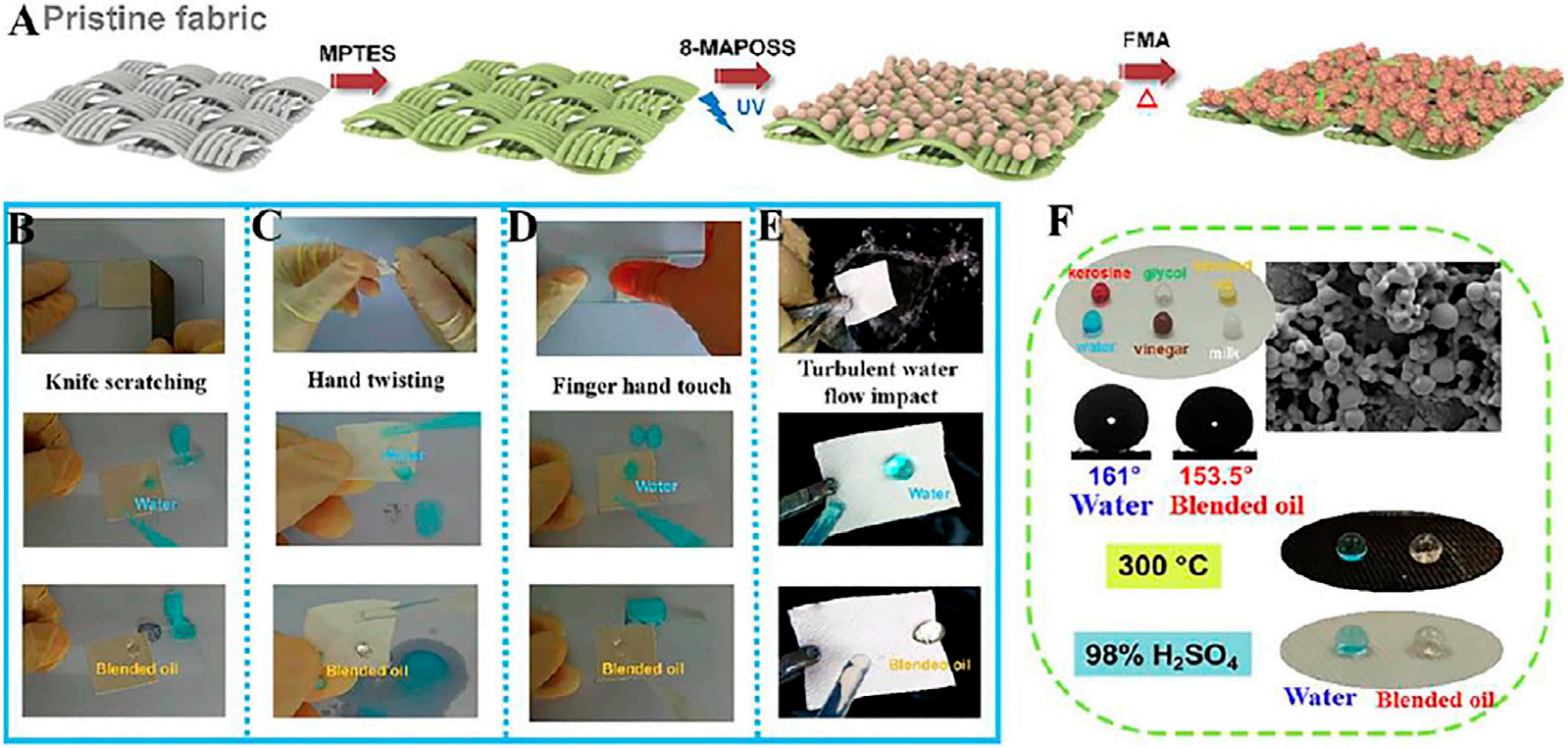
**(A)** Schematic diagram of manufacturing process of Fabric-S-MAPOSS-F; Durability tests through **(B)** knife-scratching, **(C)** hand twisting, **(D)** finger hand touch, and **(E)** turbulent water flow impact. **(F)** Common droplets (kerisine, dyed with oil red dye; glycol, colorless; blended oil, yellow; water; vinegar, brown; milk, lacte) on fabric, and liquid repellency of Fabric-S-MAPOSS-F after immersion in 98% H_2_SO_4_ for 30 min and 300°C heating for 2 h (Luo et al., 2020).

Luo et al. (Luo et al., 2020) prepared a sturdy and durable fluorinated 8-Methacryl polyhedral oligomeric silsesquioxane Cage Mixture-based superamphiphobic fabric (Fabrics-S-MAPOSS-F) (Figure 12A), which could easily float on the surface of water or mixed oil, and could resist high temperature and acid corrosion (Figure 12F). The navigation speed of Fabrics-S-MAPOSS-F in water and mixed oil is increased by 2.5 times, and the drag reduction rate is up to 154.7%. As shown in Figure 12B–E, the mechanical stability of the superamphiphobic fabric is evaluated through knife-scratching, finger hand touch, hand twisting, and turbulent water flow impact, the results show that Fabrics-S-MAPOSS-F is still superhydrophobic.

The beetle (Zhu et al., 2018c; Zhu et al., 2019; Zhu et al., 2021e) uses the special structure of the shell to collect water to provide itself with water resources. The cactus spines have a round cone-shaped wedge structure with Laplace pressure and surface energy gradient on the surface to achieve water collection (Zhu et al., 2016b). Inspired by natural creatures, lots of superhydrophobic materials are developed for water collection (Zhang et al., 2021b; Zhu et al., 2021f).

**FIGURE 13:**
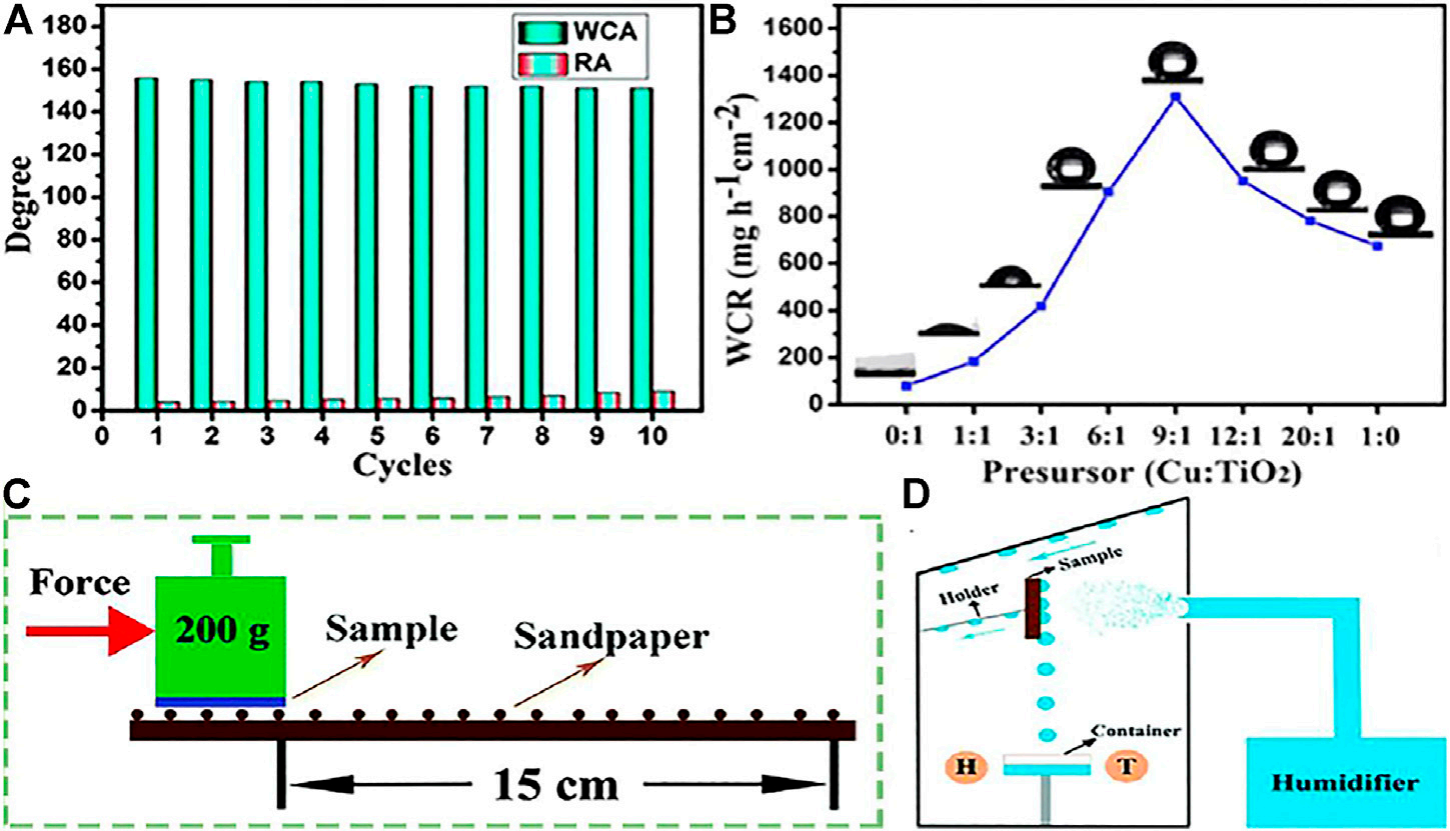
**(A)** WCA and RA on the surface after abrasion test. **(B)** Water collection rate changed with the precursor of Cu and TiO_2_. **(C)** Schematic of the abrasion test. **(D)** Schematic diagram of self-made fog collection system, H and T represent the humidity thermometer (Zhu and Guo, 2016a).

Zhu et al. (Zhu and Guo, 2016a) used copper particles and titanium dioxide particles to prepare coatings with superhydrophobic properties which can be used for water collection (Figure 13D). As shown in Figure 13B, when the molar ratio of the prepared sample precursor is 9:1, the water collection rate is the biggest water collection rate of 1309.9 mg h^−1^ cm^−2^, and showed an approximate WCA and RA of 155.11, 4.51, respectively. After sandpaper friction (Figure 13C), it is observed that there is no great change in WCA and RA (Figure 13A) due to the excellent adhesion of epoxy resin is helpful to improve the surface firmness, indicating that the coating has excellent mechanical wear resistance.

## Conclusion

Superhydrophobic materials with outstanding mechanical and chemical stability are highly vital in practical application. This review elaborates the progress of mechanical–chemical superhydrophobic materials in recent years. Firstly, the typical superwetting models are introduced, such as “Young’s contact,” “Wenzel,” “Cassie,” “Wenzel–Cassie,” “Lotus,” and “Gecko” model. Secondly, some mechanical–chemical superhydrophobic models and corresponding tests to evaluate mechanical and chemical durability are discussed. Finally, the application of these mechanical–chemical superhydrophobic materials is described. Although great scientific progress has been made in the research of durable superhydrophobic surfaces, up to now, almost no superhydrophobic surface can withstand all types of wear required by strict industrial requirements and commercial standards. Therefore, the following are some of our views and opinions:

(1) There are a great many studies to increase the mechanical properties of superhydrophobic materials, and there are many differences in the durability tests carried out. However, unified standards to measure the durability of superhydrophobic materials are lacking and should be formulated.
(2) At present, the durable superhydrophobic surface has not been widely employed in practical application, which indicates that the development of durable superhydrophobic surface should take practical application into consideration.
(3) In the preparation of superhydrophobic materials, many used organic materials are harmful to the human body and environment. Environment-friendly materials and green preparation technology are highly recommended.

We believe that a comprehensive and depth review will provide strategic guidance for the development of multifunctional durable superhydrophobic materials, and that the most challenging aspect is to create a durable superhydrophobic material without affecting wettability. We believe that a comprehensive review can provide new ideas for the development and application of superhydrophobic materials. The research of durable superhydrophobic materials is constantly developing and innovating, and its research will become a hot development direction in the next few years.
